# Comparative analysis of gastric emptying between patients undergoing EUS-guided gastroenterostomy and enteral stent placement: Pilot analysis

**DOI:** 10.1055/a-2586-6153

**Published:** 2025-05-16

**Authors:** Sridhar Sundaram, Ameya Puranik, Akhil Mahajan, Kiran Mane, Rahul Puri, Aditya Kale, Prachi Patil, Shaesta Mehta

**Affiliations:** 1 221116 Department of Digestive Diseases and Clinical Nutrition, Tata Memorial Hospital, Mumbai, India; 2 221116 Department of Nuclear Medicine, Tata Memorial Hospital, Mumbai, India; 3 29549 Department of Gastroenterology, Seth GS Medical College and KEM Hospital, Mumbai, India

**Keywords:** Endoscopy Upper GI Tract, Dilation, injection, stenting, Malignant strictures, Endoscopic ultrasonography, Intervention EUS

## Abstract

**Background and study aims:**

Previous studies show that endoscopic ultrasound-guided gastroenterostomy (EUS-GE) is better than enteral stents (ESs) in terms of clinical success and need for reintervention in the long term. Gastric atony and stent placement across a tumor can lead to poor gastric emptying after ES placement. We aimed to compare gastric emptying across ES and EUS-GE to assess the benefit of EUS-GE over ES in the short-term.

**Patients and methods:**

In this pilot study, patients who underwent ES or EUS-GE for palliation of gastric outlet obstruction were included. A gastric emptying study was performed 2 weeks after the procedure after consumption of a semisolid test-meal (Indian porridge) labelled with Tc-Sulphur. The primary outcome was gastric emptying t1/2 between ES and EUS-GE.

**Results:**

Forty patients were included in this study (21 ES, 19 EUS-GE; mean age 54 years, 27 males). EUS-GE had a faster emptying t1/2 as compared with ES (72 mins vs 118 mins) ( *P* = 0.02). Abnormal emptying was seen in fewer patients undergoing EUS-GE (5.3% vs 47.6% [ES]; *P* = 0.004). Clinical success at 2 weeks and reintervention at 6 months was no different.

**Conclusions:**

EUS-GE was associated with better gastric emptying compared with ES, suggesting a benefit that may translate into clinical benefit even in the short term.

## Introduction

**Fig. 1:**
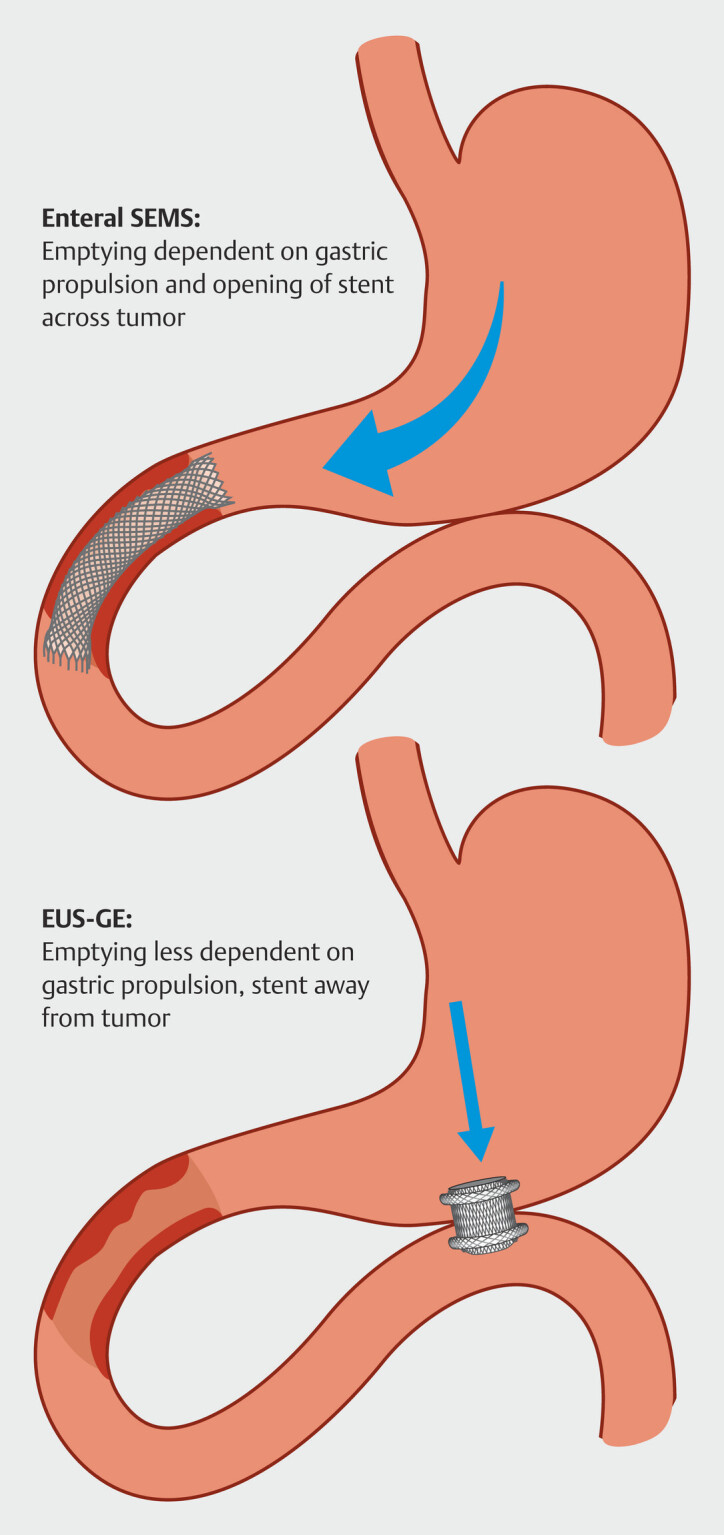
Graphic depiction of gastric emptying in patients undergoing ES and those undergoing EUS-GE.

Enteral stents (ESs) are commonly used for palliation of gastric outlet obstruction (GOO), with low morbidity and high success. The clinical course, however, is often complicated due to recurrent obstruction caused by tumor infiltration 1 . Endoscopic ultrasound-guided gastroenterostomy (EUS-GE) involves insertion of a lumen apposing metal stent from the stomach to the small bowel distal to the obstruction 2 . Previous series have shown better outcomes with EUS-GE as compared with ESs in terms of reintervention 3 . However, no previous comparison is available to assess benefit in the short term. Gastric propulsion may drive symptomatic benefit after use of an ES unlike EUS-GE, in which the stent is placed more proximally with less dependence on gastric motility to propel food. We hypothesized that ESs are likely to have lower clinical success as compared to EUS-GE due to inadequate opening of the stent placed across the tumor and gastric atony leading to poorer emptying even in the short term ( Fig. 1 ). We aimed to compare gastric emptying in patients undergoing EUS-GE and placement of an ES in the short term as a pilot.

## Patients and methods

### Study population

In this retrospective observational study, patients who were scheduled to undergo either ES placement or EUS-GE for palliation of unresectable malignant GOO between April 2022 and April 2023 were screened from the prospectively maintained endoscopy database (OIEC/4483/2024/00001). Patients who underwent ES with WallFlex enteral stents (Boston Scientific Ltd., USA; 22-mm diameter) or EUS-GE using 20×10 mm Hot Axios stents (Boston Scientific Ltd., USA) and a gastric emptying study were included. Exclusion criteria were previous surgery for GOO or previous enteral surgery, poor performance status (Eastern Cooperative Oncology Group performance status > 2) or other critical illness leading to hospitalization.

### Study interventions

Baseline demographic details, site and nature of malignancy, site of GOO, and baseline GOO symptom score (GOOSS) were noted. ES placement and EUS-GE were performed as per patient discretion and both choices were offered to patients using standardized patient information. ES placement and EUS-GE were performed by an experienced endoscopist proficient in advanced endoscopic intervention. On follow-up at 2 weeks after ES placement or EUS-GE, patients were assessed for ability to take oral feeding and GOOSS was charted. Patients underwent a gastric emptying study after consumption of a semisolid test meal (Indian porridge [Upma] 100 gm) labelled with 0.8 to 1.2 mCi of Technetium-99m sulphur colloid using a dual-head gamma camera. Patients were fasted for 12 hours prior to the procedure. Readings of gastric emptying were taken at 0, 1, 2, and 4 hours and t ^1/2^ of semisolid emptying was calculated. Subsequent follow-up at 1, 3, and 6 months was reported.

### Outcome measures

The primary outcome was gastric emptying time with t ^1/2^ of a semisolid test meal. The normal value of t ^1/2^ was 120 minutes for semisolids as per previous Indian normative data 4 . Values above 120 minutes were considered as abnormal. Secondary outcomes were the proportion with abnormal emptying, clinical success (2-point increase in GOOSS at baseline and at 2 weeks), and need for reintervention at 6 months.

## Results

During the study period, 68 patients underwent EUS-GJ or ES, of whom 40 patients who underwent gastric emptying studies were included. Nineteen of them underwent EUS-GJ and 21 underwent enteral stenting. All patients were deemed surgically inoperable due to the underlying disease burden. Baseline characteristics are listed in Table 1 .

**Table:** **Table 1** Baseline parameters.

	Enteral SEMS (n = 21)	EUS-GE (n = 19)	*P* value
Age (yrs)	53.33 (±14.9)	55.0 (±13.4)	0.712
Male: female	12:9	15:4	0.186
Baseline performance status	ECOG PS 1: 14	ECOG PS 1: 11	0.567
	ECOG PS 2: 7	ECOG PS 2: 8	
Charlson Comorbidity index (median [range])	6 (6–9)	7 (6–9)	0.855
Primary malignancy	Gallbladder: 5	Gallbladder: 5	0.174
	Stomach: 8	Stomach: 3	
	Pancreas/periampullary: 6	Pancreas/periampullary: 10	
Site of obstruction (in relation to ampulla)	Type 1: 14	Type 1: 13	0.529
	Type 2: 7	Type 2: 5	
	Type 3: 0	Type 3: 1	
Baseline GOOSS	Score 0: 3; Score 1: 18	Score 0: 9; Score 1: 10	0.038
Presence of ascites	6 (28.5%)	3 (15.7%)	0.457
ECOG PS, Eastern Oncology Group performance status; EUS-GE, endoscopic ultrasound-guided gastroenterostomy; GOOSS, gastric outlet obstruction symptom score; SEMS, self-expanding metal stent.			

**Fig. 2:**
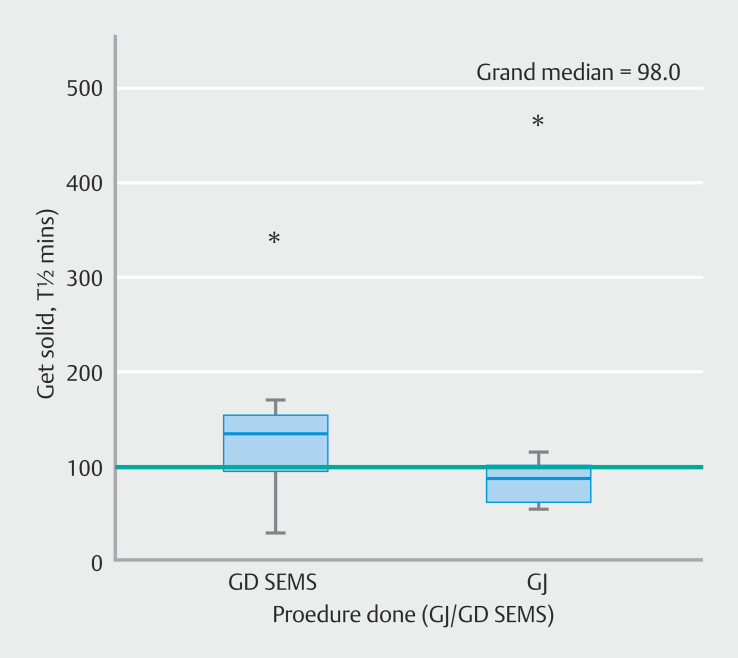
Comparison of medians of gastric emptying t1/2 in patients undergoing EUS-GE and ES.

Median gastric emptying t ^1/2^ of semisolids was significantly lower for patients who underwent EUS-GJ as compared to those who underwent ES (72 minutes; interquartile range [IQR] 57–93 vs 118 minutes; IQR 99–140; *P* = 0.021) ( Fig. 2 ). Abnormal emptying was seen in a significantly greater number of patients with ESs than those with EUS-GJ (10 of 21 [47.6%] vs 1 of 19 [5.3%]; *P* = 0.004; odds ratio [OR] 9.04, 95% confidence interval [CI] 1.27–64.21). Clinical success as defined by 2-point improvement in was achieved in 89.5% of patients (17 of 19) who underwent EUS-GJ vs 66.7% of patients (14 of 21) with ESs ( *P* = 0.08). Ability to continue oral intake did not differ between the groups. On follow-up at 6 months, reintervention was needed in three patients who underwent ES placement as compared with one patient who underwent EUS-GE over 6 months ( *P* = 0.67).

## Discussion

Most comparative studies between EUS-GE and ES placement have assessed outcomes in the long-term 5 6 . The present study shows that patients who undergo EUS-GE have better semisolid gastric emptying than patients undergoing placement of ESs in the short term, with significantly more ES patients having abnormal emptying. Chandan et al, in a meta-analysis of 659 patients from five retrospective studies comparing outcomes of EUS-GE and ES, showed comparable technical success (EUS-GE 95.2%, ES 96.9%), clinical success (EUS-GE 93.3%, ES 85.6%), with higher reintervention rates with ES placement (EUS-GE 4% vs ES 23.6%) 7 . In a recent randomized trial, Teoh et al showed that need for reintervention was much lower in patients undergoing EUS-GE as compared with ESs (4% vs 29%, *P* = 0.0002) 8 . However clinical success was defined by a 1-point increase in GOOSS, which was not significantly different at 3 days. Those who underwent EUS-GE had better GOOSS at 1 month as compared with ESs. This may be attributable to poorer emptying of gastric contents at 1 month.

Atony is known to occur in patients with long-standing GOO. In addition, because the ES is placed across the tumor, there may be inadequate opening of the stent. These factors may contribute to objectively poorer emptying 9 . Although technical success is known to be high for ESs (> 97%), clinical success is achieved in fewer patients (87%). Hence augmenting oral intake to solids may be difficult in patients undergoing ES placement, impacting nutrition and clinical outcomes. On the other hand, a recent systematic review reported the pooled adverse event rate for EUS-GE to be approximately 13% with high rates of technical success (> 90%) and higher clinical success (96%) 10 .

Our pilot study has the advantage of documenting objective outcome of gastric emptying t1/2 measured in the short term on follow-up in patients undergoing EUS-GE or ES placement. The limitations are the retrospective nature of the research, small sample size, single-center data, and non-assessment of nutritional parameters.

## Conclusions

To conclude, EUS-GE is associated with better semisolid emptying as compared with ES in the short term, which may translate into better clinical success in the short and long term for these patients. EUS-GE should be considered the primary modality for palliation of GOO for eligible patients in centers that have expertise with better emptying of gastric contents to ensure nutritional optimization.
